# Nanoelectronic Detection of Acetone with MIL-53(Al)−NH_2_ Metal–Organic Framework on Single-Walled Carbon Nanotubes

**DOI:** 10.1021/acsami.4c16016

**Published:** 2024-11-21

**Authors:** Samia Afrin, Zidao Zeng, Ganesh Kesavan, Wenting Shao, Yiwen He, Nathaniel L. Rosi, Alexander Star

**Affiliations:** † Department of Chemistry, University of Pittsburgh, Pittsburgh, Pennsylvania 15260, United States; ‡ Department of Chemical and Petroleum Engineering, University of Pittsburgh, Pittsburgh, Pennsylvania 15260, United States; § Department of Bioengineering, University of Pittsburgh, Pittsburgh, Pennsylvania 15261, United States

**Keywords:** metal−organic frameworks, single-walled carbon nanotubes, MIL-53, sensors, acetone detection, volatile organic compounds, chemiresistor, fluorescence

## Abstract

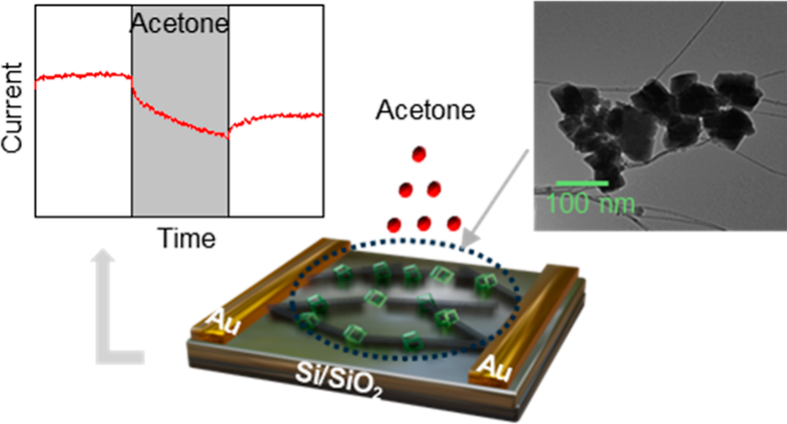

A new composite material has been synthesized by incorporating an amine-functionalized MIL-53 (Al) metal–organic framework (MOF) and single-walled carbon nanotubes (SWCNT) under hydrothermal conditions. This hybrid material combines the porosity of the MOF with the electrical conductivity of SWCNT. The preservation of MIL-53(Al)–NH_2_ MOF structure and morphology in the composite with SWCNT was verified by X-ray diffraction, Fourier transform infrared spectroscopy, scanning electron microscopy, transmission electron microscopy, and Brunauer–Emmett–Teller surface area analysis. A characteristic current–voltage (*I*–*V*) curve showed the electrical conductivity of this composite and gave a linear response in the chemiresistive sensor with increasing concentrations of acetone. The MIL-53(Al)–NH_2_/SWCNT composite also displayed fluorescence quenching tendencies across various acetone concentrations, likely attributable to the impact of guest molecules influencing the framework. An alternative approach utilizing layer-by-layer sensor device fabrication was employed for growing this MOF on carbon nanotubes directly onto a silicon chip, demonstrating its potential for versatile on-chip-sensing applications.

## Introduction

Metal–organic frameworks (MOFs) are a class of permanently porous inorganic–organic coordination polymers, which exhibit massive structural and chemical diversity, a variety of pore sizes, large-surface area, and are amenable to a wide range of synthetic techniques.^1^ Due to their great structural properties and tunable chemical characteristics, MOFs have shown promising features to be used in different environmental applications, sensing, gas storage, and separation.^2−5^ However, their direct application as electrochemical sensors is challenging because of the poor conductivity of most MOFs. Most organic ligands used in MOF synthesis are unable to support electron transfer in the framework.^6^ This limits the application of MOFs in some cases, where electrical properties are desired. Therefore, effective strategies are needed to leverage the remarkable characteristics of MOFs while addressing these challenges in sensing applications.

Carbon nanotubes (CNTs), on the other hand, have excellent electrical properties and have been widely used for chemical sensing. CNTs have exceptionally high-surface area and composed almost entirely of surface atoms which make them highly sensitive toward changes in their chemical environment.^7^ However, there are some basic challenges that need to be solved to make a chemical sensor from CNTs. The impact of humidity and temperature on the CNT sensors is notably significant, potentially leading to errors in precise measurements.^8^ Polymer functionalization can enhance selectivity but restricts surface access to the nanotubes, thereby limiting the detection ability of most CNT-based sensors to analytes with substantial binding energies.^9−12^ Due to high porosity and easy functionalization of MOFs, they can be combined with different materials and functional groups.^4^ The combinations of MOFs and carbon nanotubes have been shown to form nanocomposites that combine a high electrical conductivity of carbon nanotubes and high absorption capacity of MOFs.^13^ In such nanocomposites, CNTs establish pathways characterized by rapid electron-transfer rates and ion diffusion, thereby facilitating electrochemistry-associated application of MOFs.^14,15^ Incorporating single-walled carbon nanotubes (SWCNTs) into MOFs offers an alternate structure for enhancing conductivity, ensuring minimal pore disturbance, or imparting supplementary traits to nonconductive frameworks while harnessing synergistic effects.^12^

Within the realm of MOFs, the MIL-53 series stands out due to their adaptable framework structures and robust chemical and thermal stability.^16,17^ MIL-53 (Al) consists of chains of corner-sharing AlO_6_ octahedra which are interconnected through functionalized terephthalate units, forming a three-dimensional network.^18^ MIL-53(Al)–NH_2_, which incorporates 2-aminoterephthalate linkages, holds particular interest for applications like gas separation, gas storage, catalysis, and host guest chemistry as it has potential for enhanced selectivity or greater storage capacity due to their porous framework structures.^19,20^ Among several MOFs, MIL-53(Al)–NH_2_ also exhibits outstanding fluorescence attributes due to ligand-to-metal charge transfer (LMCT) effects.^21^ MIL-53(Al)–NH_2_ with Co@CNT has been synthesized for application of the removal of bisphenol AF (BPAF) and metribuzin from wastewater with improved adsorption and selectivity.^22^ Carbon nanotubes can also confer electrical conductivity to this specific MOF, thereby broadening its practical applications while maintaining its chemical stability.

In this study, we synthesized composites consisting of MIL-53(Al)–NH_2_ and SWCNTs and investigated their potential applications in chemical sensing. The composites were synthesized by growing MIL-53(Al)–NH_2_ MOF on the surface of commercial SWCNTs with 3 atomic % oxygen-containing functional groups. Carboxyl groups on the sidewalls and termini of SWCNTs reacted with the MOF precursors to form a composite with a beads-on-a-string morphology which is uniquely suited for chemical sensing applications.^12,23−25^ Various characterization techniques were employed to confirm the preservation of the MOF’s crystal structure in the synthesized composite with SWCNTs, thus validating the functionalization process. In this work, we explored the use of the porous structure of a nonconductive MIL-53(Al)–NH_2_ MOF to form a conductive composite for the detection of volatile organic compounds (VOCs) at room temperature. Previous studies have shown that this MOF exhibits strong host–guest interactions with acetone molecules.^17,18^ Therefore, we investigated the sensitivity of the synthesized composite to varying concentrations of acetone, a key organic solvent that is used extensively in various industries. Despite its wide usage, acetone is toxic and poses significant health risks. Especially, acetone as a common indoor air pollutant necessitates continuous monitoring for environmental health concerns with a maximum allowable concentration set at 250 ppm.^27,28^ Although many commercial acetone sensors are available, most are oxide-based and require high operating temperatures. Room-temperature sensors focus on detecting lower concentrations (ppb or lower ppm levels), yet exposure to acetone concentrations between 300 and 500 ppm can cause irritation and nausea in adults.^26^ The synthesized composites were integrated into silicon chips with interdigitated gold electrodes and tested as chemiresistive sensors to analyze their responses to varying concentrations of acetone. The chemical interactions of the composite with acetone molecules were also investigated spectroscopically by observing alterations in the composite’s fluorescence to understand the mechanism of detecting acetone at the interface of the prepared MIL-53(Al)–NH_2_/SWCNT composite. We have also investigated the direct growth of this MOF on the SWCNT networks deposited in between interdigitated gold electrodes on a silicon chip under hydrothermal synthesis conditions.

## Results and Discussion

### Synthesis Route and Characterizations

**Figure 1:**
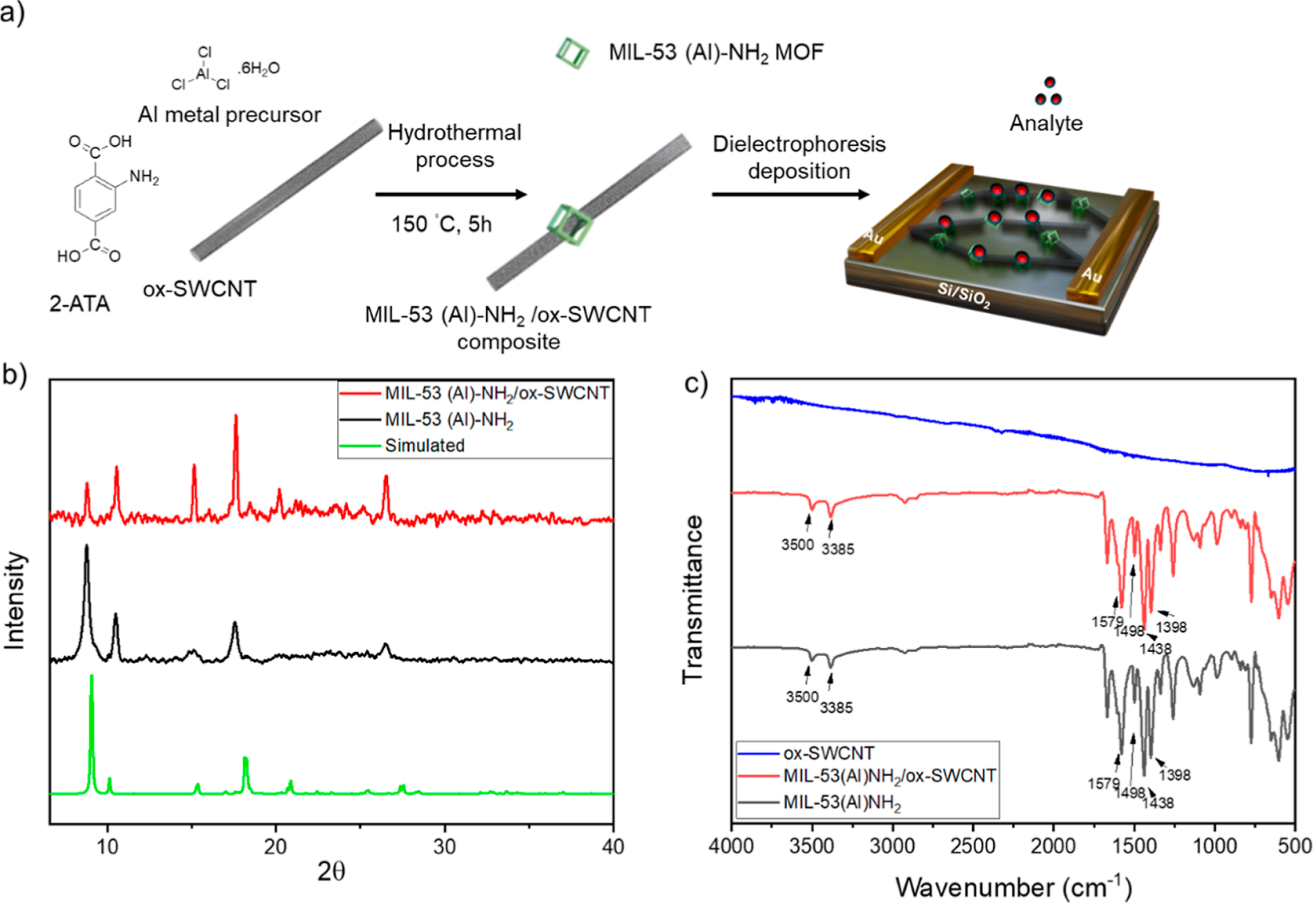
(a) Synthesis of MIL-53(Al)–NH_2_/ox-SWCNT composite and fabrication of chemiresistive sensors from the composite. (b) Simulated XRD pattern of amine-functionalized MIL-53 (Al), experimental XRD pattern of amine-functionalized MIL-53 (Al) MOF and MIL-53 (Al)–NH_2_/ox-SWCNT composite; and (c) FTIR spectra of ox-SWCNT, MIL-53 (Al)–NH_2_/ox-SWCNT composite, and MIL-53 (Al)–NH_2_ MOF.

The synthesis of MIL-53(Al)–NH_2_ composite with SWCNTs was executed under hydrothermal conditions (Figure 1a). To attain uniform dispersion in aqueous solution and facilitate chemical reaction between MOF and nanotubes, oxidized carbon nanotubes (ox-SWCNT) were used.^24^ Ultrasonic bath sonication (135 W) of ox-SWCNTs in water (1 h) was performed to promote the breakdown of large carbon nanotube agglomerates into smaller bundles and individual ox-SWCNTs, facilitating dispersion. The hydrothermal synthesis procedure of the MIL-53 (Al)–NH_2_ MOF was followed from a published method with slight modifications.^19^ It was anticipated that by deferring the deprotonation of the MIL-53 (Al)–NH_2_ MOF precursor, a slower nucleation rate of MOF might achieve favorable interaction on the surface of the SWCNT. The MOF precursors can interact with carboxylic groups on the sidewalls of ox-SWCNTs, resulting in the formation of covalent bonds. These bonds can act as nucleation sites, facilitating the growth of the MOF structure. Alternatively, ligands may noncovalently interact with sidewalls of ox-SWCNT through pi–pi stacking, serving as templates for MOF growth.^12,23−25^ This reaction mechanism is based on room-temperature synthesis with step-by-step addition of either metal or linker precursors of different MOFs with SWCNTs.

The synthesis under hydrothermal conditions necessitated additional exploration of the relevant pathways and favorable interactions while precursors were mixed together to yield these heterostructure composites. Our previous studies have shown that direct mixing of ox-SWCNT with MOFs does not effectively functionalize the MOFs on the sidewalls of ox-SWCNTs. This approach has been unsuccessful in forming a conductive carbon nanotube network with MOFs for the preparation of conductive devices.^23^ We also conducted optimization experiments using various wt % of ox-SWCNT loading in the composite (Figure S1) and selected the optimal composition of 1.5 wt % ox-SWCNT (Figure S1b) in the composite for all sensing experiments. At lower nanotube loadings, the MOFs tend to overgrow the nanotubes (Figure S1a), preventing the formation of conductive devices. Conversely, at higher nanotube loadings (Figure S1c), there is insufficient MOF to provide effective binding sites for analyte detection.

In this synthesis, water was used as a solvent to delay deprotonation of 2-amino terephthalic acid (2-ATA). This MOF crystal tends to aggregate instead of forming monocrystal when deprotonation becomes faster.^29^ Slowing down the deprotonation can promote the heterogeneous nucleation, while ox-SWCNTs are also present in the solution. Using water as a solvent, when deprotonation of 2-ATA molecules was delayed, we speculate that Al clusters and the sidewalls of SWCNTs can interact via covalent interaction and MOF can grow from the Al clusters interacting with SWCNT which can favor the heterogeneous nucleation forming MIL-53(Al)–NH_2_/ox-SWCNT composite.

Powder X-ray diffraction (XRD) was conducted to confirm the crystal structure of the amine-functionalized MIL-53/ox-SWCNT composite, as shown in Figure 1b. The XRD pattern of the composite was in good agreement with that of the simulated and synthesized MOF structure. Characteristic diffraction peaks at 8.7, 10.4, 15.1, 17.5, and 26.4^°^ were observed which correspond to the (101), (200), (202), and (020) planes.^30^ A small shift from the locations of the simulated XRD peak was noticed which might be due to the functionalization of CNTs to the MOF framework.^17,31^ A change in intensity was observed in the composite compared with the MOF, likely due to crystal orientation effects induced by CNTs. Previous reports have shown that intensity variations can occur when this MOF forms a composite with CNTs, suggesting that relative intensities may vary between different samples.^16,32^ However, because the diffraction pattern closely matched that of MIL-53(Al)–NH_2_ and previous studies,^22,30^ we conclude that the MOF structure is preserved within the composite.

Infrared spectroscopy was used as a complementary technique to evaluate the MOF structure upon the addition of nanotubes. As shown in Figure 1c, comparison of ox-SWCNT, MOF, and the composite was observed. The peaks in ox-SWCNT were less pronounced compared to the MOF and, in the composite, the peaks were aligned with the MOF structure. The peaks at 3500 and 3385 cm^–1^ correspond to amino groups stretching vibration which were similar to the 2-ATA ligand (Figure S2).^20^ This similarity indicated that amino groups present in the composite or MOF did not participate in coordination.^33^ Vibrational bands in the region of 1398–1579 cm^–1^ were assigned to the carboxylic acid functional groups of Al coordination in the MOF structure. Bands at 1398 and 1438 cm^–1^ can be attributed to carbonyl symmetric stretching, and 1498 and 1579 cm^–1^ bands can be assigned to carbonyl asymmetric stretching.^29^ The broad peak in the range of 2300–3300 cm^–1^ for the precursors (Figure S2) has almost disappeared, which can be due to the elimination of the –OH group of the carboxyl group from the linker. The observed spectra for both the MOF and the composite matched the previous reports,^29,30,33^ indicative of minimal effects of incorporation of ox-SWCNT on the overall MOF structure.

**Figure 2:**
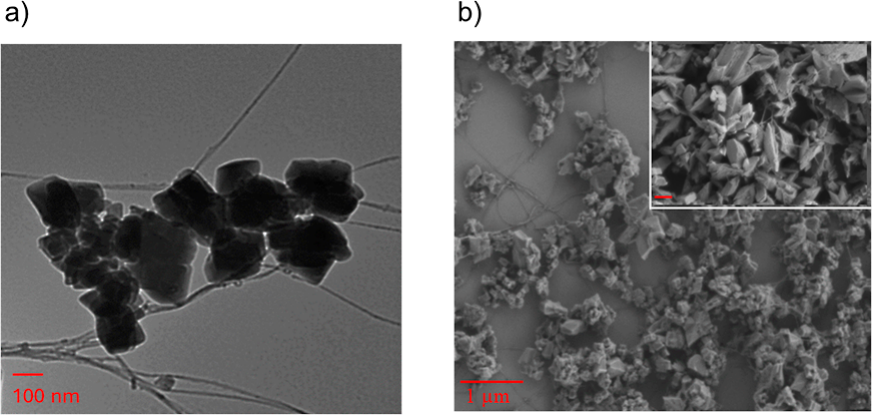
(a) Transmission electron microscopy (TEM) and (b) scanning electron microscopy (SEM) images of MIL-53(Al)–NH_2_/ox-SWCNT composite, with high magnification inset SEM image of the composite coated with PdAu alloy (scale bar 300 nm).

The morphology of the prepared composite was investigated by using transmission electron microscopy (TEM) and scanning electron microscopy (SEM) (Figure 2a,b). The microscopy images reveal the successful synthesis of MOF crystals on sidewalls of ox-SWCNTs with exposed nanotube surfaces. No free MIL-53 (Al)–NH_2_ crystals were observed, which are not associated with nanotubes. Three dimensional rhomboid crystals are seen in Figure 2b which matches with previous reports^21,29^ to confirm the synthesis of MOF crystals with an approximate size of 200–300 nm.

**Figure 3:**
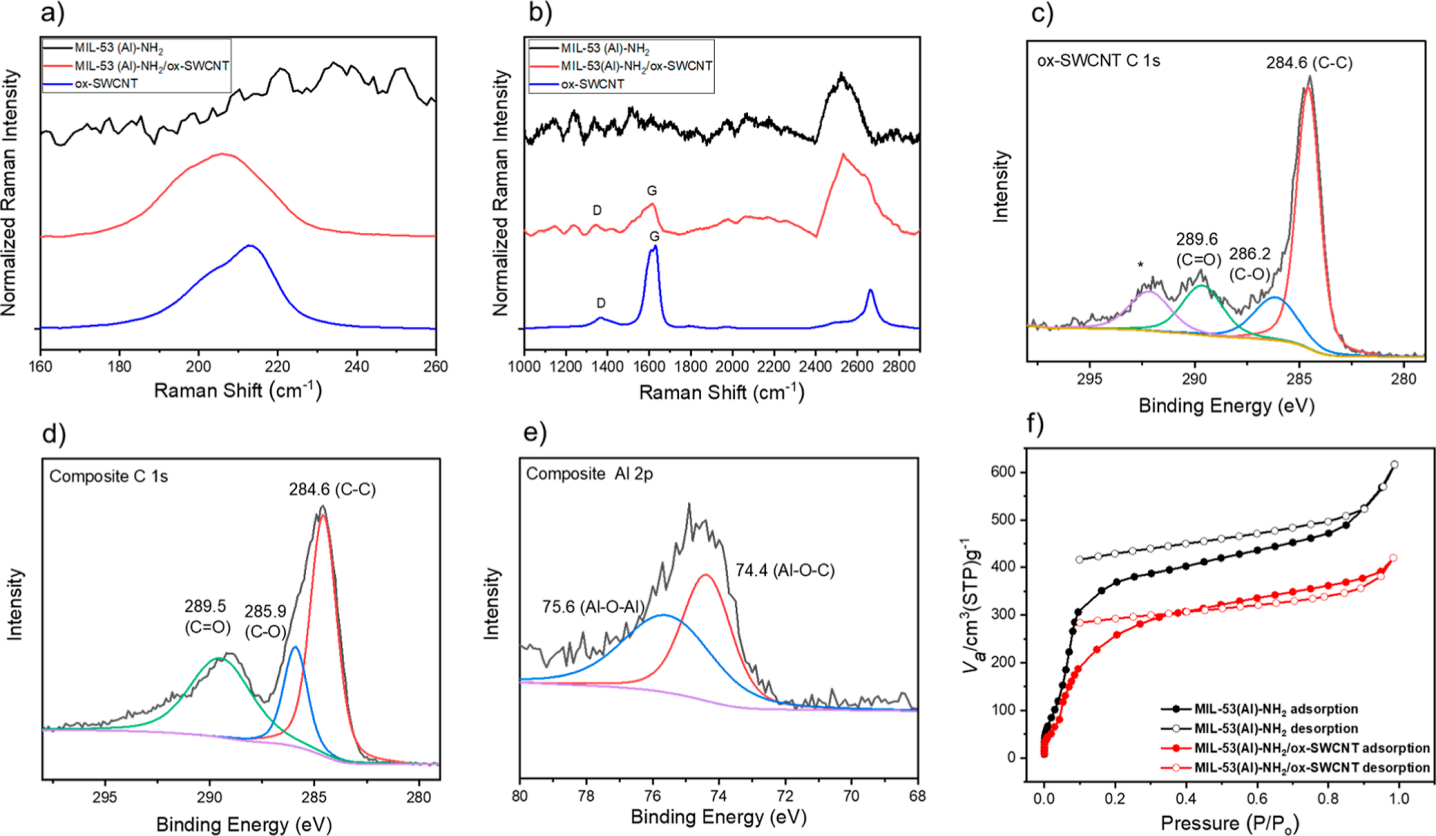
Raman spectra (a,b) for ox-SWCNT, MIL-53 (Al)–NH_2_/ox-SWCNT composite, and MIL-53 (Al)–NH_2_ MOF; (a) RBM region (785 nm excitation laser); (b) D and G peak region (638 nm excitation laser). All spectra were normalized. High-resolution XPS spectra (c–e) for ox-SWCNT and MIL-53 (Al)–NH_2_/ox-SWCNT composite. (c) C 1s (asterisk represents a satellite peak) spectra of ox-SWCNT. (d) C 1s and (e) Al 2p spectra of the composite with deconvolutions of the overall signal. (f) N_2_ adsorption and desorption isotherms of the MOF and the composite.

To inspect the functionalization between the MIL-53 (Al) MOF and SWCNTs, characterization by Raman spectroscopy and X-ray photoelectron spectroscopy (XPS) was performed for ox-SWCNTs and the composite. Raman spectra showed a shift in the radial breathing mode (RBM). The RBM peak is associated with the diameter distribution of SWCNTs.^34^ RBM shifting to lower wavenumbers, i.e., from 212 to 205 cm^–1^ (Figure 3a), is indicative of the large MOF structures attachment to the defects sites of ox-SWCNTs during their functionalization with MIL-53 (Al)–NH_2_. The ratio between intensities of D and G peaks reflects degree of defects on ox-SWCNTs.^35^ An observed increase (from 0.13 to 0.31) in *I*_D_/*I*_G_ (Figure 3b) corresponds to increase in defects degree which also indicate functionalization of ox-SWCNTs with the MOF. The peaks in the range of 2500–2600 cm^–1^ are characteristic peak of 2D band for ox-SWCNT, MOF, and composite, the peak in the region of around 2600 cm^–1^ might be associated with presence of μ–OH moieties in the framework with strong intermolecular hydrogen bonding.^12,36,37^ XPS scans were also performed for both samples (Figure 3c–e, Table S1). The shifting of binding energy of C–O of the composite compared to bare ox-SWCNTs (Figure 3c,d) can be due to Al binding covalently to the defect sites.^23^ The intensity of this bond also increased in the composite, which indicates generation of more C–O bonds in composite which can be associated with presence of both MOF (C–O from the ligands) and ox-SWCNT (C–O from the defect site) in the same material. The Al–O–C bond in the composite (74.4 eV) comparing with SWCNT (Figures 3e and S3) can be from the covalent bond between MOF and the defect site of the SWCNT. However, it would be negligible compared to Al–O–C bond in the MOF itself, so these two contributions were not observed distinctly through the XPS spectra. There is one additional peak in the 292.4 eV region (Figure 3c: labeled with an asterisk) which can be denoted as a satellite peak of the larger peak in the graphitic structure.^38^ A mixture of ox-SWCNT/Al precursor XPS spectra was also taken as a control experiment (Figure S4) to understand the interaction. Al–O–Al bonding was dominant compared to the Al–O–C bond because of the larger chains of Al–O–Al of the MOF compared to the interacted site.

To examine the porosity of the MOF-nanotube composite, N_2_ sorption isotherms were collected for both the MOF alone and the composite (Figure 3f). The calculated Brunauer–Emmett–Teller (BET) surface area for the MOF was 950 m^2^/g, which is comparable to literature reports.^29,39^ The MOF-nanotube composite is also porous, exhibiting a BET surface area of 760 m^2^/g. The surface area of the composite is less than that of the pure MOF due to the presence of the carbon nanotube; this result is consistent with our previous reports on related composite materials.^12,23^

### Electrical Performance of the Composite: Acetone Detection

**Figure 4:**
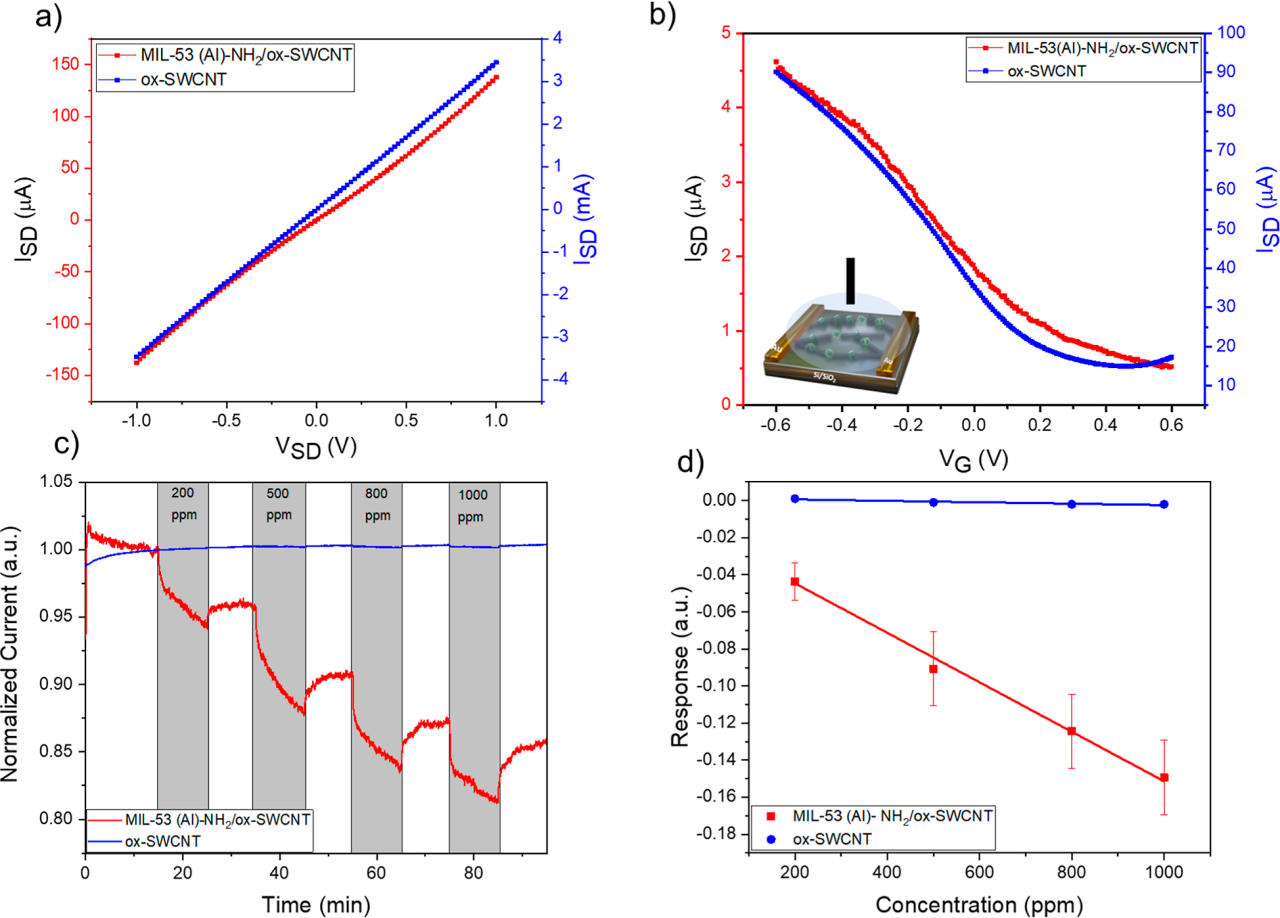
Electrical characteristics of MIL-53(Al)–NH_2_/ox-SWCNT composite. (a) Source-drain (SD) current–voltage curve for SWCNT and composite. (b) Source-drain current (*I*_SD_) versus gate voltage (*V*_G_) liquid gated FET characteristics, MIL-53 (Al)–NH_2_/ox-SWCNT composite (red) and SWCNT (blue). (c) Normalized current responses versus time, i.e., chemiresistor responses for different concentrations of acetone (200, 500, 800, and 1000 ppm) of the composite (red) and ox-SWCNT (blue); (d) Calibration curve for sensor responses for varying concentrations of acetone (error bar represents device to device variation of 3 devices).

The electrical conductivity of the synthesized composite deposited on the device (Figure S5) was confirmed by collecting current–voltage (*I*_SD_–*V*_SD_) curves (Figure 4a). The curves indicate a hybrid material that inherits the electrical conductivity of the carbon nanotubes with the nonconductive MOF. Since MIL-53(Al)–NH_2_ MOF is water stable,^21^ it appears to be well suited for fabrication of liquid-gated field-effect transistor (FET). The composite exhibits p-type semiconducting behavior in the liquid FET transfer characteristics (*I*_SD_–*V*_G_), with a current on–off ratio of 9, surpassing the on–off ratio of bare ox-SWCNTs, which is 5, as illustrated in Figure 4b. MIL-53(Al)–NH_2_/ox-SWCNT composite decorated chemiresistor devices showed a decrease in conductance after exposure to varying concentrations of acetone (Figure 4c). The calibration curve based on the acetone sensor responses is shown in Figure 4d. The chemiresistor response was calculated by measuring the relative change in the current.
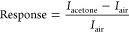
In the equation of relative sensor response above, *I*_acetone_ is the current after 10 min of acetone exposure and *I*_air_ is the current in dry air condition without any acetone exposure.

Compared to the response of ox-SWCNT for acetone (Figure 4c), the synthesized composite gives a higher response. A possible explanation for the sensing of acetone with this composite is molecular kinetic diameter (∼5 Å) match, and rhombohedral channels of this MOF are well suited to take up small polar molecules like acetone.^17,18,40^ The porous structure of the MOF accommodates the acetone molecules, and hence, a change in current is observed. The decrease in current observed upon exposure to the analyte can be attributed to the nature of the devices, which feature a network of carbon nanotubes. The interface between these tubes with this MOF may greatly influence their electrical properties. Even minor changes in the distance between the nanotubes can impact the contact resistance as the likelihood of charge tunneling diminishes exponentially with distance.^41^ Consequently, exposure to acetone may induce a change in current when the composite comes in contact with the analyte.

We initiated the experiment by testing acetone at the highest concentration (∼30 v/v %) using bubbler with a 10 min exposure period (Figure S6) during which the flow stabilized. This 10 min duration was then used as a standard for subsequent tests across lower acetone concentrations. With the highest concentration, calculated *t*_90_ value was 87 s, which was determined at the point where the sensor’s response reached 90% of its maximum for the composite. According to Langmuir adsorption model, the saturation behavior of the MOF binding sites can vary with acetone concentration due to adsorption kinetics and the interaction strength between acetone molecules and the MOF framework. At lower concentrations, saturation is slower as acetone molecules gradually occupy the binding sites, whereas at higher concentrations, binding sites fill more quickly, resulting in faster saturation—consistent with our findings. For these cases, sensing responses are also evaluated as slope responses, calculated from the absolute change in conductance within this defined exposure time.^49^

The sensing performance of a liquid-gated field-effect transistor (FET) was studied with different acetone concentrations (4, 9, 18, 27, 36, and 45 mM) (Figure S7), corresponding to the range (∼200–2600 ppm) tested in chemiresistor devices. As the acetone concentration increased, we observed a shift in the FET transfer characteristics, resulting in a decrease in the source-drain current (*I*_SD_) at a fixed gate voltage. This decrease in current is consistent with the results obtained from chemiresistive measurements, where higher acetone concentrations led to a decrease in the current (Figure 4c). The *I*_SD_–*V*_G_ curves shifted to more negative gate voltages as the acetone concentration increased, suggesting that electron transfer occurs between acetone molecules and the p-doped SWCNTs within the composite. This shift can be due to pore filling of MOFs and indicative of interactions between the acetone molecules and the MOF’s porous structure, which accommodates the acetone and impacts electrical conductivity of the composite.^12,40,41^

### Fluorescence Study: Understanding the Mechanism of Acetone Detection

**Figure 5:**
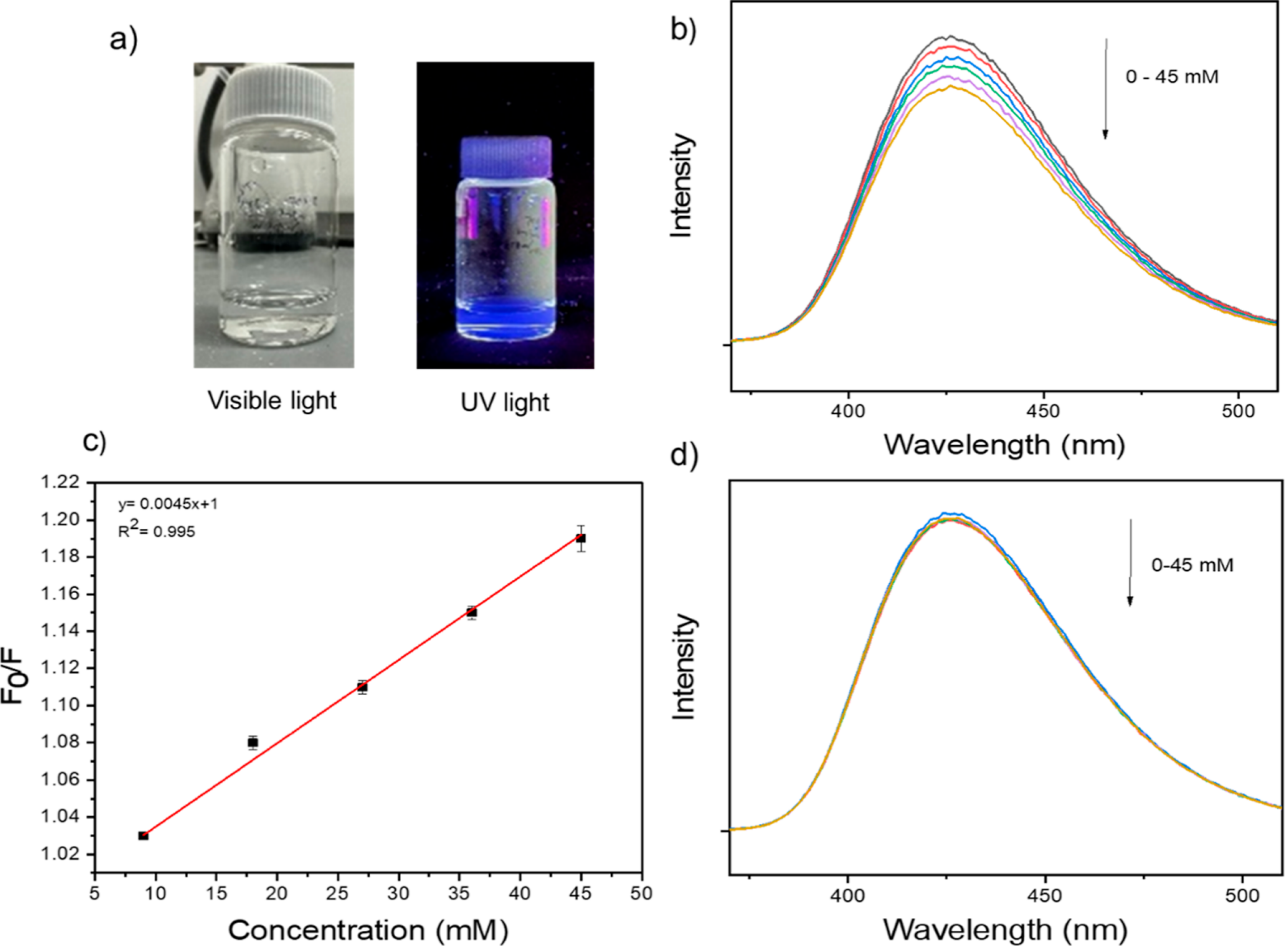
Fluorescence characteristics of MIL-53 (Al)–NH_2_/ox-SWCNT composite. (a) Aqueous suspensions of the composite under visible and UV light; (b) fluorescence emission spectra (excitation 305 nm) for different concentrations of acetone 0, 9, 18, 27, 36, and 45 mM from top to bottom sequentially; (c) corresponding Stern–Volmer fitting curve of the composite toward different concentration ranges of acetone; (d) fluorescence emission spectra for 2-ATA with acetone concentrations 9, 18, 27, 36, and 45 mM.

Due to the strong luminescence property of MIL-53 (Al)–NH_2_ MOF, optical characteristics were also assessed for the synthesized composite. Fluorescence experiments were conducted for different concentrations of acetone with this composite to further prove the influence of acetone molecules on the framework. While the electrical performance of the composite demonstrated by the change in current with increasing concentration of acetone provides insights into the conductive part of the composite, fluorescence studies can offer a deeper understanding of the role that specific MOF porous structure plays in acetone detection within the synthesized composite. The emission spectra were taken at different excitation wavelengths to explore the luminescent properties of the synthesized composite (Figure S8). Under UV light, the composite showed violet fluorescence (Figure 5a). There was a consistent strong emission peak at 426 nm which can be assigned to π to π* electron transitions of the benzene rings of the ligand component.^21^ The emission spectral analysis of the composite with acetone at different concentrations (9–45 mM) is shown in Figure 5b. It showed a continuous decrease in fluorescence (FL) intensity which can be related to quenching behavior for acetone.^42^

The efficiency of quenching (*F*_0_/*F*) was determined depending on the fluorescence of the composite in the presence and absence of acetone, which was represented by *F* and *F*_0,_ respectively. A linear relation was observed between the quenching efficiency and acetone concentrations between 9 and 45 mM in the Stern–Volmer fitting curve (Figure 5c).

Since the FL emission comes from the ligand 2-ATA, the FL experiment for the ligand with acetone was also carried out. The ligand does not show any intensity change with different concentrations of acetone (Figure 5d). This observation indicates that the porous structure of MIL-53(Al)–NH_2_ is responsible for changes in fluorescence intensity of different concentrations of acetone. Emissions are linked to the transfer of energy from ligands to metal ions. Therefore, when guest molecules enter the framework, energy transfer might be disturbed impacting the intensity of fluorescence and hence, the quenching behavior with acetone is observed in the composite.^21,43^ UV–vis absorption spectra for synthesized MOF, composite, and bare ox-SWCNTs are shown in Figure S9a. Excitation wavelength of 305 nm was used for MIL-53(Al)–NH_2_/ox-SWCNT composite during quenching, and UV–vis absorption spectrum of acetone (Figure S9b) showed no spectral overlap in this excitation region. This observation provides evidence that the influence of acetone in quenching is most likely attributed solely to the formation of the framework in the composite. The electrical data highlight the conductive behavior influenced by acetone, while the fluorescence analysis sheds light on how the porous nature of the MOF contributes to the overall sensing capabilities of the composite. Together, these studies provide a comprehensive picture of the detection mechanism of the composite.

### Selectivity, Humidity, and Stability

To evaluate the selectivity of the synthesized material, various molecules were tested based on the size of the MOF’s porous structure. Our hypothesis was that the kinetic diameter of the molecules should match with the porosity of the MOF. Initially, we compared the chemiresistive sensing performance of the larger benzene molecule (6 Å) with that of acetone at the same concentration (100 ppm) (Figure S10).^44^ Since benzene produced no response, this confirmed our hypothesis that only molecules around 5 Å in size, such as acetone, fit within the MOF’s porous structure.

To further investigate the role of the porous framework, we conducted fluorescence studies on molecules with similar sizes—formaldehyde (3.7 Å), ethanol (4.3 Å), isopropyl alcohol (4.6 Å), and methanol (3.8 Å)—all at the same concentration (1000 ppm) (Figure S11).^45,46^ Molecules of similar size elicited a response, supporting the hypothesis of specific interactions with the MOF, particularly with molecules containing carbonyl groups, which we plan to explore further in future research.

The synthesized composite deposited on chip was exposed to 100% humidity with different concentrations (100 and 200 ppm) of acetone (Figure S12a) in chemiresistive device. A decrease in current was observed when the sensor was exposed to 100% humid air from dry air; however, no response was observed in the presence of acetone. Although acetone interacts with the composite, the lack of response was consistent with our earlier findings, which indicated the need for a custom dehumidifier to detect acetone while using SWCNT under humid conditions.^47,48^ After the humidity test, the chip was left in an open environment at room temperature. Acetone response was taken under dry conditions before and after humidity exposure to the same chip (Figure S12b). Humidity did not affect the performance of the device before and after exposure to humid air which proves that it did not destroy the property of the composite. The stability of the composite was tested, showing similar acetone response results over time (Figure S13). After synthesis, the composite material was subjected to acetone testing at different concentrations, and the same material was retested six months later using identical acetone concentrations. Both the initial and after 6-month tests produced similar responses, proving strong evidence of the sensor’s excellent stability over time. This demonstrates that the composite maintains its performance even after extended periods, supporting its potential for long-term applications in sensing technologies. The limit of the detection of the chemiresistive sensor was calculated to be 28 ppm with the testing concentration range of 200–1000 ppm. The acetone response of the present work is compared to different acetone sensors in Table S2.

### Layer-by-Layer On-Chip Synthesis Approach

**Figure 6:**
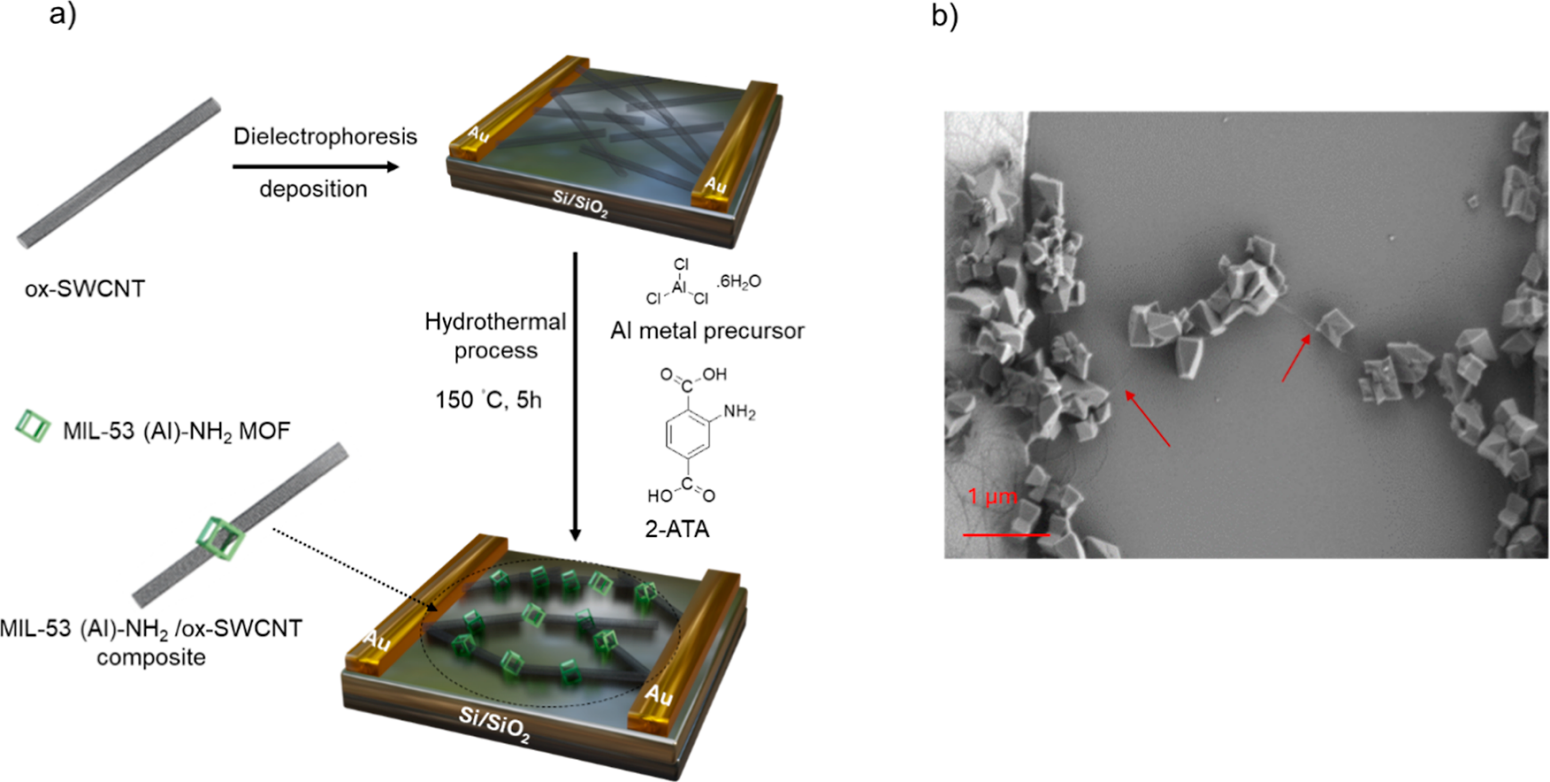
(a) Layer-by-layer synthesis route of MIL-53 (Al)–NH_2_/ox-SWCNT composite on silicon chip. (b) Scanning electron microscopy (SEM) image of MIL-53 (Al)–NH_2_ MOF grown on previously deposited ox-SWCNTs in between interdigitated gold electrodes (scale bar 1 μm).

An alternative layer-by-layer synthesis was implemented on a silicon chip, wherein the MIL-53(Al)–NH_2_ MOF can be grown on a chip with previously deposited ox-SWCNTs under analogous hydrothermal conditions (Figure 6a). MOFs were grown in between interdigitated electrodes on carbon nanotube networks (Figure 6b). Following synthesis, XRD analysis was conducted to identify the characteristic XRD peaks associated with this MOF (Figure S14) to confirm the successful synthesis of this MOF.

Electrical characterizations conducted both pre- and post-MOF growth on ox-SWCNTs and are shown in Figure S15, revealing sustained electrical conductivity before and after the MOF formation. The chip was exposed to acetone at concentrations of 200, 500, 800, and 1000 ppm, similar to those used for the synthesized composite as a chemiresistive sensor (Figure S16). Although the responses observed with this method were smaller, they remained comparable, highlighting the potential advantage in sensor fabrication through optimized nanotube and MOF deposition. This direct synthesis on chip can offer an approach to establish an efficient method of deposition and can demonstrate evaluation of sensitivity variations for a particular analyte under this specific synthesis condition.

## Conclusions

In conclusion, a new composite material of an amine-functionalized MIL-53 (Al) MOF on the surface of oxidized single-walled carbon nanotubes (ox-SWCNT) has been synthesized. The confirmation of crystallinity, functionality, and morphology of the MOF while forming composite was achieved through a comprehensive analysis involving XRD, Fourier transform infrared, SEM, and TEM. Raman and XPS spectral analyses were conducted to validate the functionalization occurring at the defect sites of ox-SWCNT. MIL-53 (Al)–NH_2_/ox-SWCNT composite effectively merged the electrical conductivity inherent in the SWCNTs with the porous characteristics of the MOF. This combination resulted in a material that showed good chemiresistive and fluorescent sensing capabilities toward different concentrations of acetone with an excellent stability of the material.

Utilizing the layer-by-layer direct synthesis approach showed better electrical characterization properties, allowing for the direct growth of the MIL-53 (Al)–NH_2_ MOF from the previously deposited ox-SWCNTs on to the chip. Our findings confirmed that only smaller molecules such as acetone effectively interact with the MOF, as evidenced by the lack of response from larger molecules like benzene. Although currently we cannot confirm the selectivity for the similar size molecules, the composite, benefiting from its high porosity and excellent electrical conductivity, holds promise of selective acetone detection by preparing sensor arrays with different MOFs in the future research focused on finding “fingerprints” for targeted analytes.

## Experimental Section

### Preparation of Ox-SWCNT Suspension

Ox-SWCNTs (P3-SWNT, Carbon Solutions, Inc.) were prepared separately in 0.1 mg/mL solution of water. The solution was sonicated for 1 h to suspend the ox-SWCNTs. The ox-SWCNT suspension was sonicated for 15 min before being used in the synthesis.

### Synthesis of MIL-53 (Al)–NH_2_/SWCNT Composite

Synthesis of MIL-53 (Al)–NH_2_ MOF was carried out following published procedure by the hydrothermal process.^19^ For composite material synthesis, the ox-SWCNT suspension was mixed with the MOF precursor solution inside an autoclave. The specific ratio and methods are provided in the Supporting Information

### Fabrication of Devices

Prefabricated 2.6 × 2.6 mm^2^ Si/SiO_2_ chips with eight interdigitated gold electrodes to form 6 μm channels were used to fabricate the devices (Figure S5) and wire bonded and potted with polydimethylsiloxane into a dual inline 40-pin ceramic package. Optical image of the device was obtained using an Olympus1 × 81/1 × 2- UCB microscope. For deposition of the composites, drop-casting and dielectrophoresis (DEP) methods were used. While drop-casting, 5 μL of the sample suspension (0.1 mg/mL) was deposited on the devices and annealed at 200 °C for drying. When DEP was used, a Keithley 3390 Arbitrary Waveform Generator was used to generate a sine wave. The conditions maintained for DEP were 10 Vpp, 1 MHz, and 4 min. The devices were washed with water after DEP and annealed at 200 °C for 1 h before use.

### Chemiresistive Sensing

For chemiresistive sensing, the material was tested in a custom-made gas flow chamber, recording the changes in current at a constant voltage of 0.05 V. Sensing experiments were carried out by first purging with dry compressed air at 1000 sccm. Afterward, the sensors were exposed to 200, 500, 800, and 1000 ppm of acetone for 10 min at increasing concentration. Mass flow controllers were used to generate these concentrations by diluting 1000 ppm of acetone balanced in air. Details of other techniques are discussed in the Supporting Information section.

## Supporting Information Available

TEM images of varying % of CNT loadings; FTIR spectra of MOF precursors; XPS spectra for ox SWCNT Al 2p spectra; ox-SWCNT/Al precursor; peak-fitted peak positions of XPS; optical images of the devices; high concentration acetone sensing; transfer characteristics of FET device and response curve for the composite upon increasing concentrations of acetone; fluorescence emission spectra of the composite with different excitation wavelengths; UV–vis absorption spectra of SWCNT, MOF, composite, and acetone; selectivity analysis for chemiresistive sensing of acetone and benzene on the composite; fluorescence study to understand selectivity of different molecules in relation to the porous structure of the composite; effect of humidity and chemiresistive response of the composite in acetone before and after exposure to humidity; stability test of the composite; XRD pattern of the MOF for layer-by-layer synthesized composite on a silicon chip and their electrical characterizations; and chemiresistive sensing result of layer-by-layer on-chip synthesis approach with different concentrations of acetone, their response curve, and comparison table for acetone sensors (PDF)

## Abbreviations

SWCNT: single-walled carbon nanotubes
Ox-SWCNT: oxidized single-walled carbon nanotubes
CNTs: carbon nanotubes
VOCs: volatile organic compounds
MOF: metal–organic framework
2-ATA: 2-amino terephthalic acid
