# Supplementary material for: Nanoelectronic Detection of Acetone with MIL-53(Al)−NH2 Metal–Organic Framework on Single-Walled Carbon Nanotubes
Source: ACS Appl Mater Interfaces. 2024 Nov 21;16(48):66377–86. doi: 10.1021/acsami.4c16016 (PMC11622181; doi:10.1021/acsami.4c16016)
Supplement: Supplementary file 1 — am4c16016_si_001.pdf [file am4c16016_si_001.pdf]

Supporting Information

Nanoelectronic Detection of Acetone with MIL-53(Al)−NH_2 Metal-Organic Framework on Single-Walled Carbon Nanotubes

Samia Afrin[1], Zidao Zeng[1], Ganesh Kesavan[1], Wenting Shao[1], Yiwen He[1], Nathaniel L. Rosi[1,2], and Alexander Star[*1,3]

[1]Department of Chemistry, University of Pittsburgh, Pittsburgh, Pennsylvania 15260, United States

[2]Department of Chemical and Petroleum Engineering, University of Pittsburgh, Pittsburgh, Pennsylvania 15260, United States

[3]Department of Bioengineering, University of Pittsburgh, Pittsburgh, Pennsylvania 15261, United States

[*]Corresponding Author. Email: astar@pitt.edu

TABLE OF CONTENTS

Experimental Details……………………………………………………………….……..S4

Figure S1. TEM images of the composites with varying ox-SWCNT loading ……………………...…………………………………………………………………………S8

Figure S2. FTIR spectra of MOF precursors…………………………………………………S9

Figure S3. Aluminum 2p X-ray photoelectron spectrum of ox-SWCNT………………………S10

Figure S4. X-ray photoelectron spectra of ox-SWCNT/Al precursor. a) Carbon 1s and b) aluminum 2p spectra………………………………………………………………………S10

Table S1. Peak-fitted peak positions of XPS…………………………………………………..S11

Figure S5. Optical microscope images of the interdigitated gold electrodes composite………………….……………………………………………………………...S11

Figure S6. High concentration acetone sensing with chemiresistor using a bubbler….……………….…………………………………………………………...S12

Figure S7. Acetone sensing with field-effect transistor (FET) fabricated from MIL-53 (Al)-NH_2/ox-SWCNT composite.…………………………………………………………...S12

Figure S8. Emission spectra of MIL-53 (Al)-NH_2/ox-SWCNT composite……………………S13

Figure S9. UV-Vis absorption spectra of a) SWCNT, MIL-53 (Al)-NH_2 MOF, and MIL-53 (Al)-NH_2/SWCNT composite and b) acetone……………………………………………………..S13

Figure S10. Selective chemiresistive sensing of acetone vapors versus benzene vapors (100 ppm) using MIL-53 (Al)-NH_2/ox-SWCNT composite……………………………………………….S14

Figure S11. Change in fluorescence emission of MIL-53 (Al)-NH_2/ox-SWCNT composite with different analytes (1000 ppm)……………………………………………………………..S15

Figure S12. Effect of humidity on chemiresistive sensing of acetone with MIL-53 (Al)-NH_2/ox-SWCNT composite……………………………………………………………………………..S15

Figure S13. Long-term stability test for MIL-53 (Al)-NH_2/ox-SWCNT composite for chemiresistive acetone sensing………………………………………………………………S16

Figure S14. XRD peaks for MIL-53 (Al)-NH_2 MOF after the layer-by-layer on chip direct synthesis……………………………………………………………………………………...S17

Figure S15. Electrical characterization of layer-by-layer synthesis of MIL-53 (Al)-NH_2 MOF on deposited ox-SWCNT on silicon chip...S18

Figure S16. Chemiresistive sensing of acetone with layer-by-layer synthesized MIL-53 (Al)-NH_2 MOF on silicon chip with ox-SWCNTs………………………………………………………..S18

Table S2. Comparison table for acetone sensors………………………………………………S19

References……………………………………………………………………………………..S19

Experimental Details

Chemicals. Ox-SWCNT (P3-SWNT, Carbon Solutions, Inc), aluminum chloride hexahydrate ($AlCl_3 \cdot 6H_2O$, Sigma-Aldrich), 2-aminoterephthalic acid (2-ATA, Alfa Aesar), N,N-dimethylformamide (DMF, Fisher Scientific) were purchased and used without further purification.

Synthesis of MIL-53(Al)-NH_2/SWCNT composite. To synthesize the composite by hydrothermal process, 10-mL Teflon lined stainless steel autoclave was used. To prepare amine functionalized MIL-53 and single-walled carbon nanotube (SWCNT) composite, 2-ATA and Al metal precursors ($AlCl_3 \cdot 6H_2O$) were mixed in 1:1 molar ratio. To that solution, 2 mL of 0.1 mg/mL carbon nanotube solution (optimized 1.5 wt% of the composite) was added. The precursor solutions were sonicated for 1h before mixing them together. The autoclave was heated at 150 ºC for 5 hrs. The solvent used for this synthesis was water. The resulting grey colored material was centrifuged out at 3400 rpm and washed with water and DMF for three times, filtered (0.2 µm PTFE membrane filter) and vacuum dried for 8 hrs. To remove the unreacted precursors from the pores of the MOF, the material was further treated with DMF at 150 ºC under stirring conditions. The DMF molecules were then removed by heating at 150 ºC and the resultant final product was filtered and vacuum dried and stored in water.

Synthesis of MIL-53(Al)-NH_2 MOF. The MOF was prepared using similar hydrothermal synthesis method. 2-ATA and $AlCl_3.6H_2O$ precursors were mixed in the molar ratio of 1:1 and sonicated for 1h before being combined and placed inside a Teflon lined steel autoclave. The autoclave was heated at 150 ºC for 5 hrs and resulting yellow product was obtained. The material

was centrifuged at 3400 rpm, washed 3 times with water and DMF, filtered through a 0.2 μm PTFE membrane filter, and vacuum dried for 8 hrs. Unreacted precursors were removed by heating with DMF at 150 ºC and the final product was then filtered, vacuum dried and stored in water for further characterization.

Layer-by-layer on-chip synthesis approach. For the layer-by-layer synthesis approach, 0.1 mg/ml of ox-SWCNT dispersed in DMF was deposited onto the silicon chip through dielectrophoresis (DEP). Once deposited, the chip was taken out from the package and placed inside an autoclave containing the MOF precursor solution. The synthesis on the chip proceeded in the same manner as described previously for the MOF preparation. After the synthesis, the chip was again heated with DMF at 150 ºC inside the autoclave. Finally, the chip was wire bonded for the subsequent sensing experiments.

X-ray Diffraction. Powder X-ray diffraction pattern was evaluated using Bruker D8 XRD system equipped with LynxEye detector. Each sample was dropcast on a glass slide before measuring. 2θ angles between 5º and 40º were measured at 0.02º intervals with a rate of 0.3 seconds/point. The X-ray source was Cu Kα held at 40 kV and 40 μA with a 0.2 mm aperture slit width.

Fourier Transform Infrared (FTIR) Spectroscopy. FTIR spectroscopy was measured using Perkin Elmer Spectrum 2 instrument at a resolution of 400-4000 cm^{-1}.

Transmission Electron Microscopy (TEM). TEM images were taken using FEI Morgagni (80 KeV) and Hitachi (HT7800). Samples were prepared by dropcasting 7 μL of diluted samples in water on TEM sample grids (carbon film, 400 mesh copper grid; Electron Microscopy Sciences).

Scanning Electron Microscopy (SEM). SEM imaging was performed with Zeiss Sigma 500 VP Scanning Electron Microscope. Coated sample was prepared using Denton sputter coater (20 mA, 20 s).

X-ray Photoelectron Spectroscopy (XPS). XPS data were generated on a Thermo ESCALAB 250 Xi XPS using monochromated Al Kα X-rays as the source. A 650 μm spot size was used, and the samples were charge-compensated using an electron flood gun.

Raman Spectroscopy. Raman spectra were recorded with the XplorA Raman-AFM/TERS system. The radial breathing mode (RBM) region was recorded using a 785 nm (100 mW) excitation laser operating at 1% power. D and G peaks region was recorded using a 638 nm (24 mW) excitation laser operating at 1% power.

N_2 Sorption Isotherm. Gas sorption isotherms were collected on a Micromeritics 3-flex gas adsorption analyzer. Approximately 100-120 mg of each sample was exchanged with dichloromethane (DCM) 3 times a day for 3 days to remove DMF. Samples were then degassed at 30 °C for 15 h on a Micromeritics SmartVacPrep under vacuum. After that, the samples were heated to 200 °C for 15 h under vacuum to remove remaining DCM and DMF. A liquid N_2 bath was used for the N_2 adsorption experiments at 77 K. Ultra-high purity grade N_2 (99.999%) was used for the tests.

Limit of detection calculation. Limit of detection for the chemiresistive sensor was calculated using the formula, LOD = 3δ/S, where δ denotes the standard deviation of the baseline current for 1 minute before the exposure to acetone and S represents the slope of the linear region of the calibration plot. From the baseline current, δ was found to be 0.0012 and slope from the calibration plot was 1.3×10^{-4}. Therefore, LOD was calculated to be 28 ppm.

Bubbler experiment setup. For the high concentration acetone testing setup, 99% acetone was placed inside a bubbler equipped with Teflon tubing. The sensor was exposed to acetone vapor for 10 min with the outlet Teflon tubing clamped directly above the packaged silicon chip. Initially, the chip was purged with air, followed by 10 min of exposure to acetone vapor, and then a 10 min recovery period. The concentration for the acetone vapor (~30 v/v%) was calculated assuming saturated vapor pressure under standard conditions.

FET Measurements. FET devices were investigated through liquid-gated FET device configuration by Keithley Source Meter Unit 2400. A 1 M Ag/AgCl electrode (CH Instruments, Inc.) was used as the gate electrode and PBS (blank) was used as the gating electrolyte. Acetone concentration ranging from 4 mM to 45 mM were prepared in PBS solution and tested from lowest to highest concentration. FET measurements were performed using 300 μl of PBS or different concentration of acetone in PBS as liquid gating media. In FET measurements, the gate voltage (V_G) was swept from +0.6 to −0.6 V with a source-drain voltage (V_{SD}) of 50 mV. After each measurement devices were rinsed with DI water and blown dry with nitrogen before the next gating liquid is added. Device responses were calculated using the equation $R = (I- I_0)/I_0$ at $V_G = -0.3$ V, where I_0 is the I_{SD} at −0.3 V in blank (baseline), and $I- I_0$ is the difference between I_{SD} of each acetone sample and the blank at −0.3 V. To perform the hysteresis testing, the gate voltage was also swept back from -0.6 V to +0.6 V, none of the samples showed significant hysteresis behavior.

Fluorescence Experiments. All Fluorescence spectra were recorded using HORIBA FluoroMax-3 instrument. MIL-53(Al)-NH_2/ox-SWCNT composite suspension was prepared with a concentration of 0.005 mg/ml and sonicated for 2 min before carrying out the experiment. To a 1

cm × 1 cm quartz cell, fluorescence emission wavelength vs intensity was measured for different concentrations of acetone in the suspension.

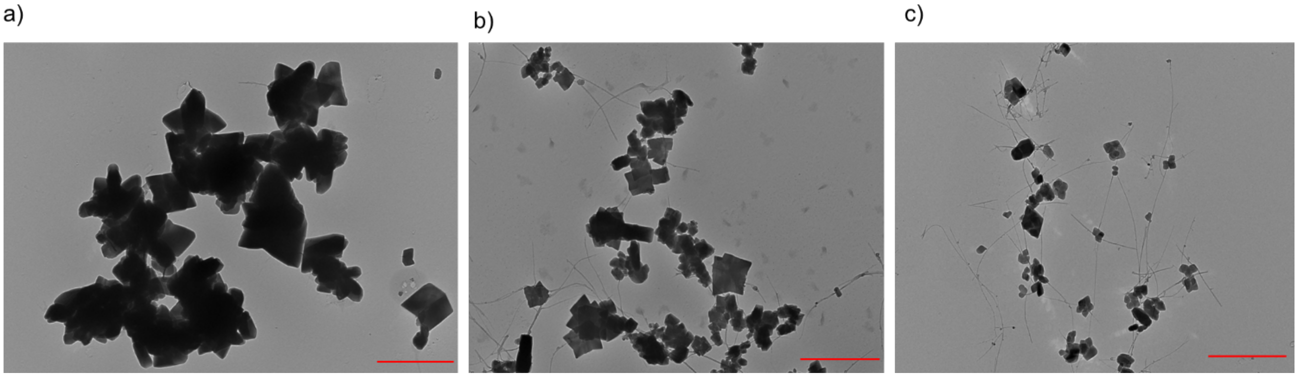

Figure S1. TEM images of the composites with varying ox-SWCNT loading a) 0.85 wt%, b)1.5 wt% (optimum), and c) 3.1 wt%. All scale bars are 1 μm.

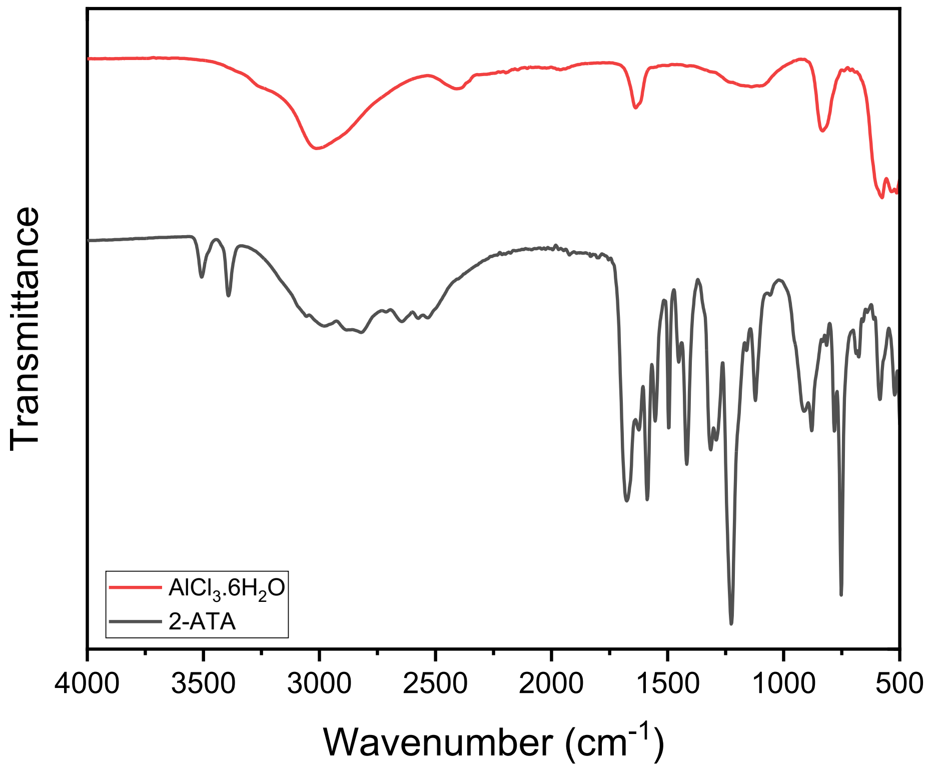

Figure S2. FTIR spectra of MOF precursors.

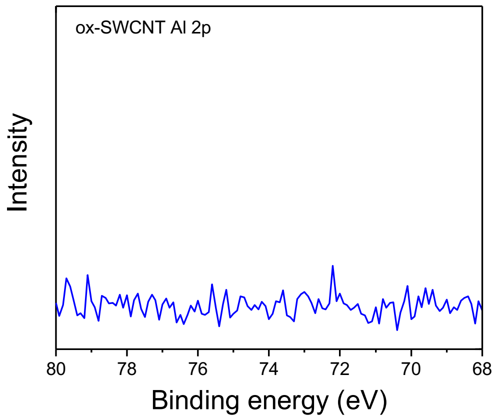

Figure S3. Aluminum 2p X-ray photoelectron spectrum of ox-SWCNT.

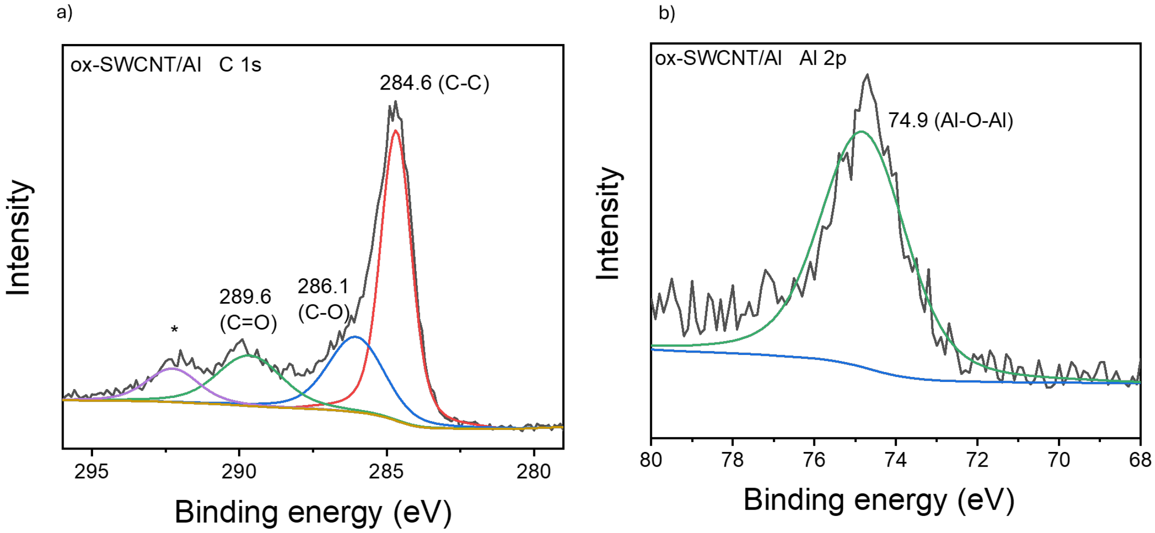

Figure S4. X-ray photoelectron spectra of ox-SWCNT/Al precursor. a) Carbon 1s and b) aluminum 2p spectra.

Table S1. Peak-fitted peak positions of XPS.

Sample name	Binding energy (eV)				
	C-C	C=O	C-O	Al-O-Al	Al-O-C
Ox-SWCNT	284.6	289.6	286.2	-	-
MIL-53 (Al)-NH_2/ox-SWCNT	284.6	289.5	285.9	75.6	74.4
Ox-SWCNT/Al	284.6	289.6	286.1	74.9	-

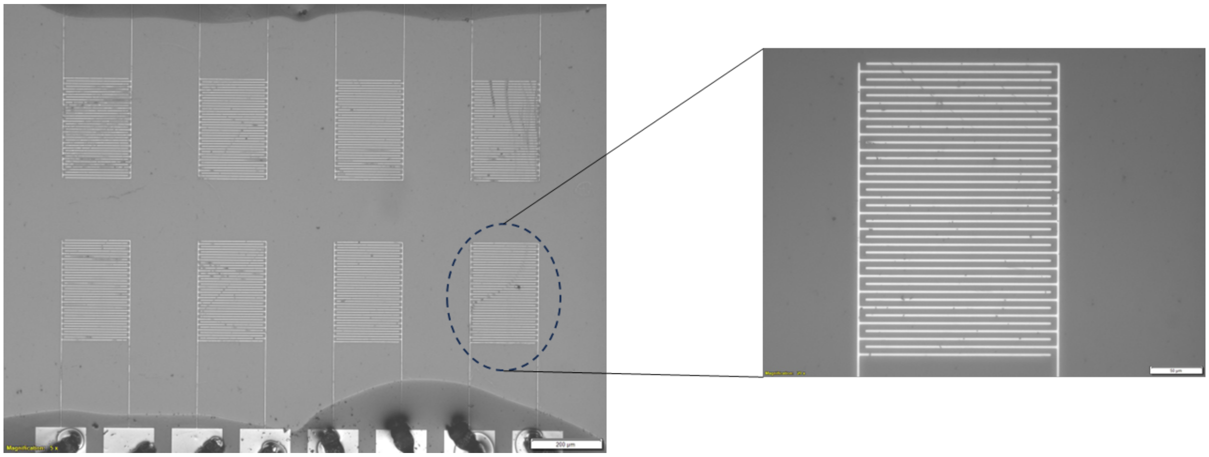

Figure S5. Optical microscope images of the interdigitated gold electrode devices used for sensing experiments.

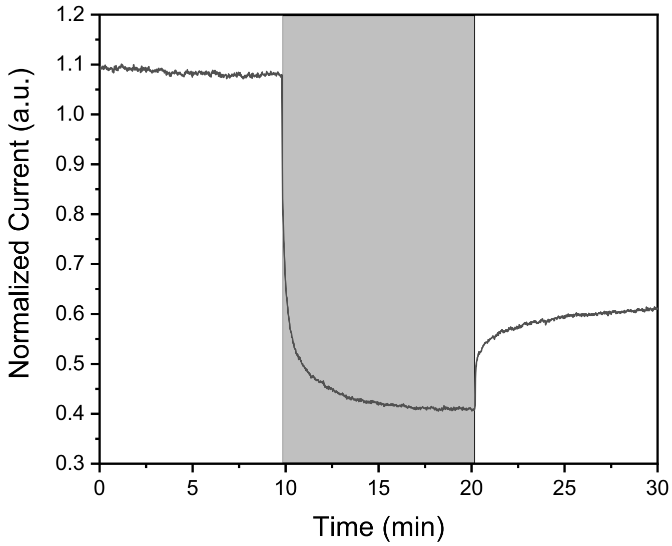

Figure S6. High concentration (30 v/v%) acetone sensing with chemiresistor using a bubbler for MIL-53 (Al)-NH_2/ox-SWCNT composite.

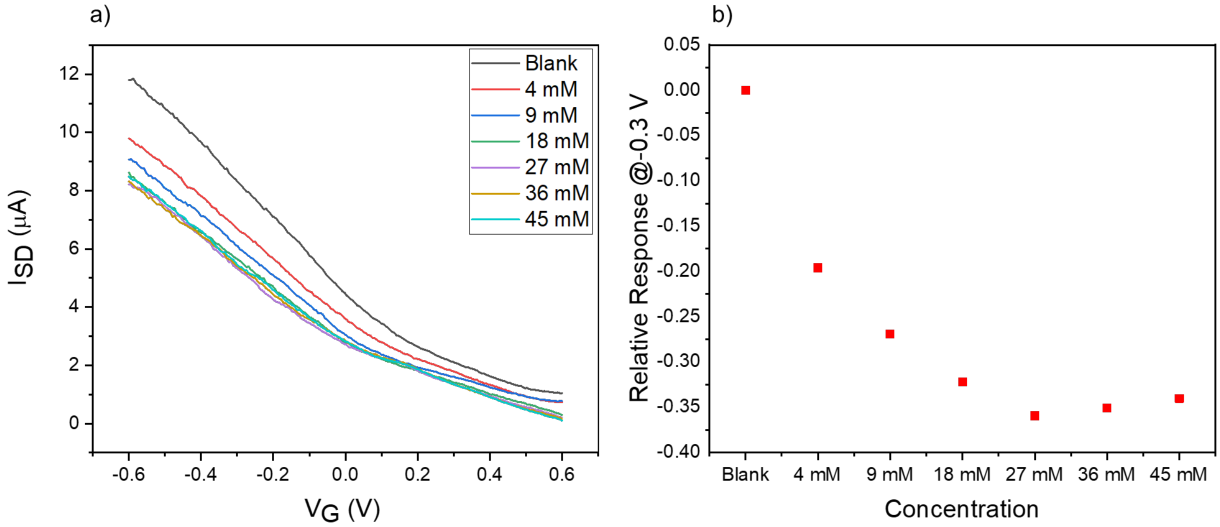

Figure S7. Acetone sensing with field-effect transistor (FET) fabricated from MIL-53 (Al)-NH_2/ox-SWCNT composite. a) Transfer characteristics, i.e., source-drain current (I_{SD}) versus liquid gate voltage (V_G), of the FET device upon addition of increasing concentrations of acetone from 4 mM to 45 mM. b) Device response, defined as a relative change in source-drain current ($I-I_0/I_0$) at –0.3 V gate voltage, for different concentrations of acetone.

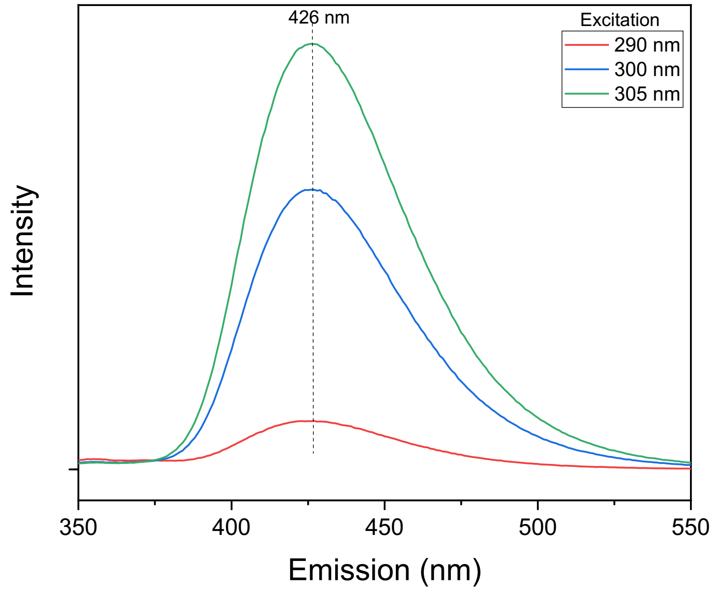

Figure S8. Emission spectra of MIL-53 (Al)-NH_2/ox-SWCNT composite. Constant emission wavelength at 426 nm was observed with different excitation wavelengths at 290, 300, and 305 nm.

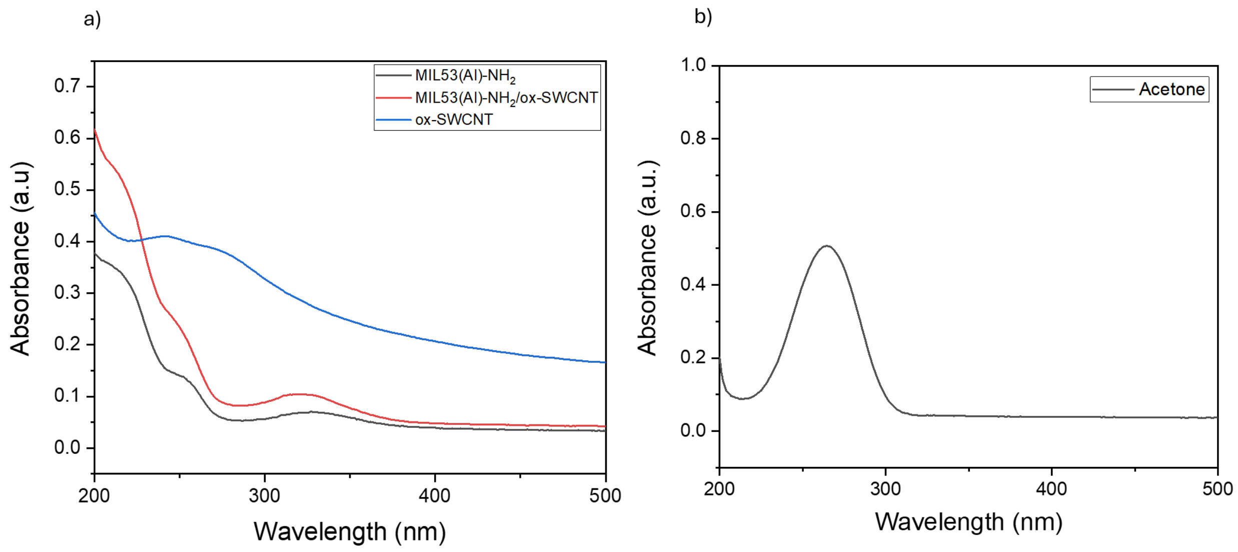

Figure S9. UV-Vis absorption spectra of a) ox-SWCNT, MIL-53 (Al)-NH_2 MOF, and MIL-53 (Al)-NH_2/ox-SWCNT composite and b) acetone.

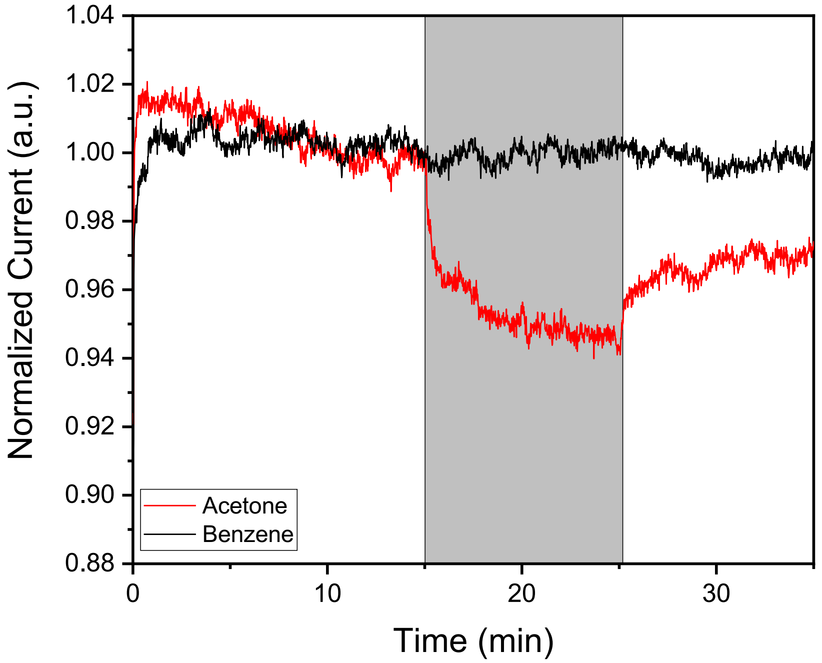

Figure S10. Selective chemiresistive sensing of acetone vapors versus benzene vapors (100 ppm) using MIL-53 (Al)-NH_2/ox-SWCNT composite.

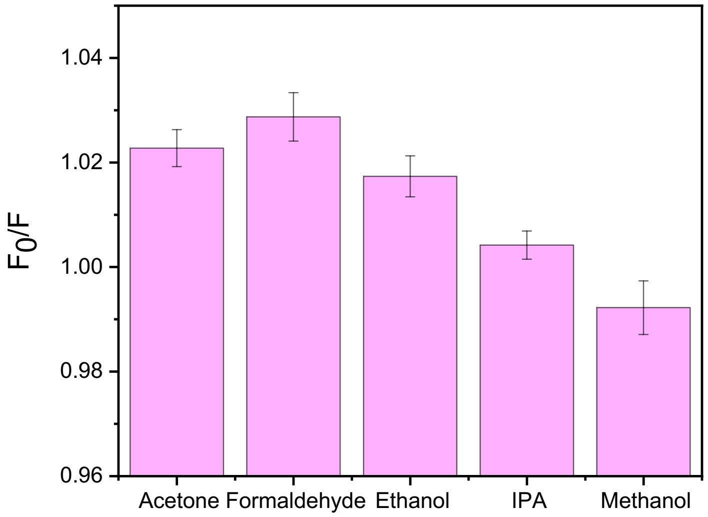

Figure S11. Change in fluorescence emission of MIL-53 (Al)-NH_2/ox-SWCNT composite with different analytes (1000 ppm). This study was conducted to understand the acetone selectivity of the composite in relation to the MOF's porous structure.

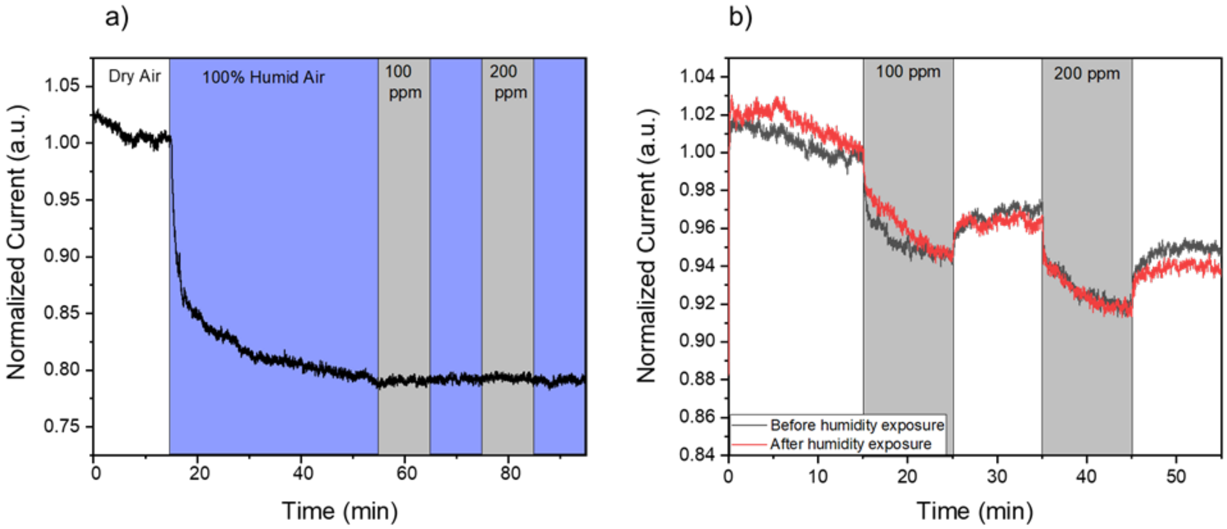

Figure S12. Effect of humidity on chemiresistive sensing of acetone with MIL-53 (Al)-NH_2/ox-SWCNT composite. a) Dry air versus humid air. b) Device response to acetone vapors (100 and 200 ppm) before and after exposure to humidity.

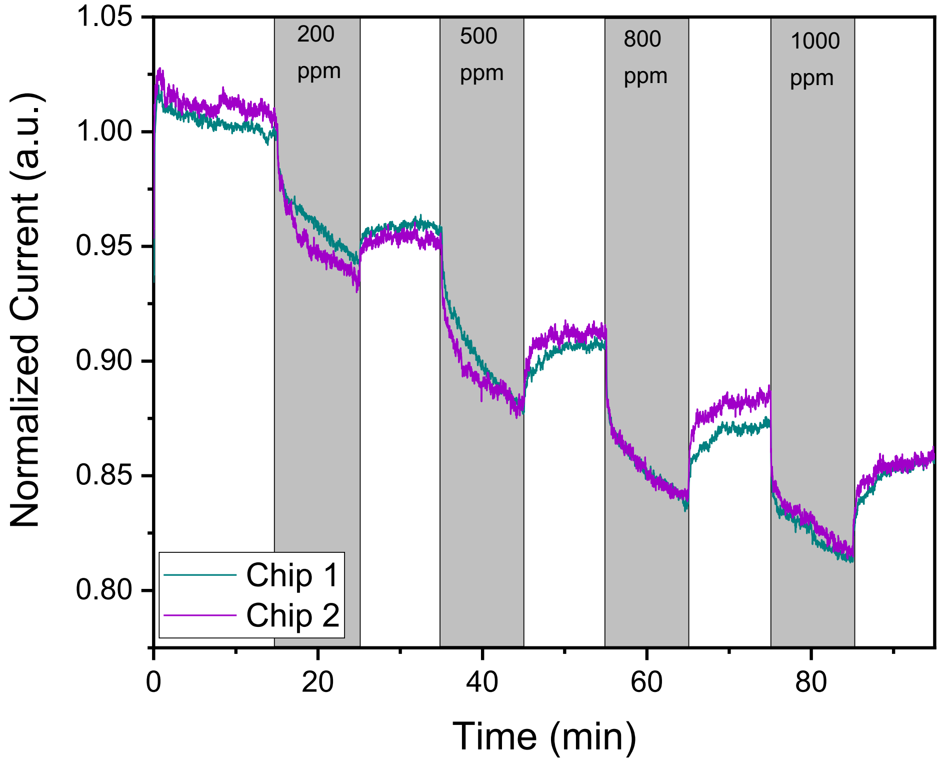

Figure S13. Long-term stability test for MIL-53 (Al)-NH_2/ox-SWCNT composite for chemiresistive acetone sensing. Chemiresistor sensor chip 1 (freshly prepared composite) and chip 2 (from the same composite after 6 months of storage) were tested for different acetone concentrations.

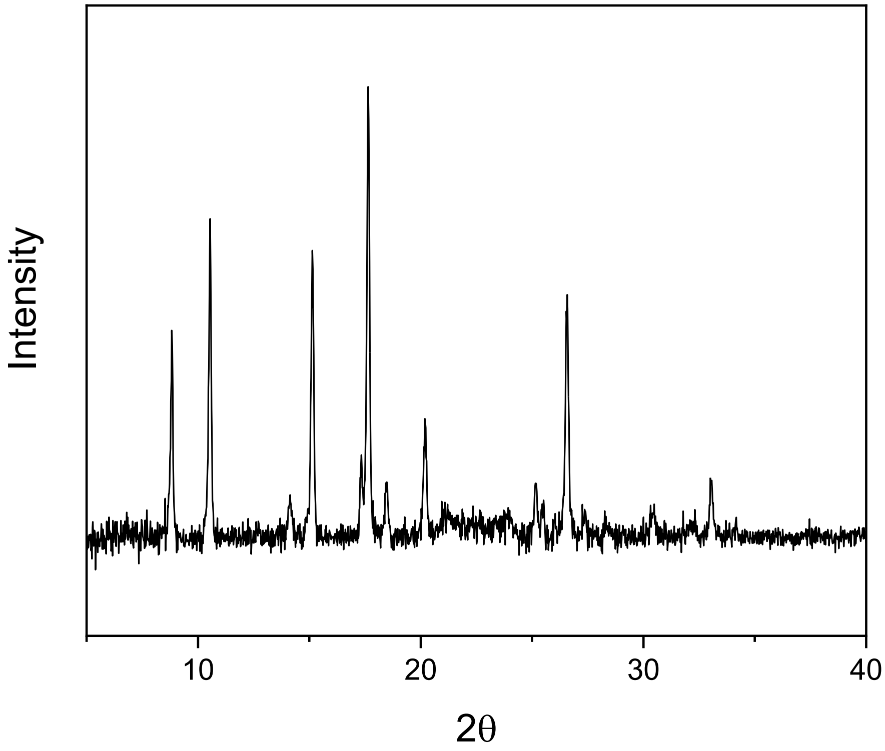

Figure S14. XRD peaks for MIL-53 (Al)-NH_2 MOF after the layer-by-layer on chip direct synthesis.

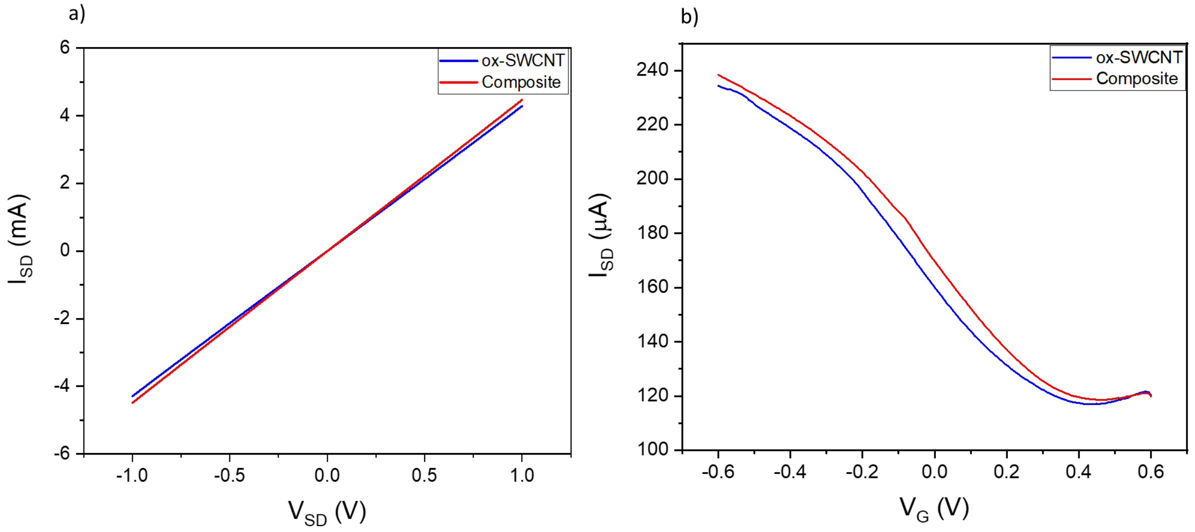

Figure S15. Electrical characterization of layer-by-layer synthesis of MIL-53 (Al)-NH_2 MOF on deposited ox-SWCNT on silicon chip. a) Characteristic source-drain current (I_{SD}) versus source-drain bias (V_{SD}) curve for ox-SWCNT and MIL-53 (Al)-NH_2/ox-SWCNT composite. b) Liquid gated FET transfer characteristics, i.e., source-drain current (I_{SD}) versus gate voltage (V_G); ox-SWCNT (blue) and the composite (red).

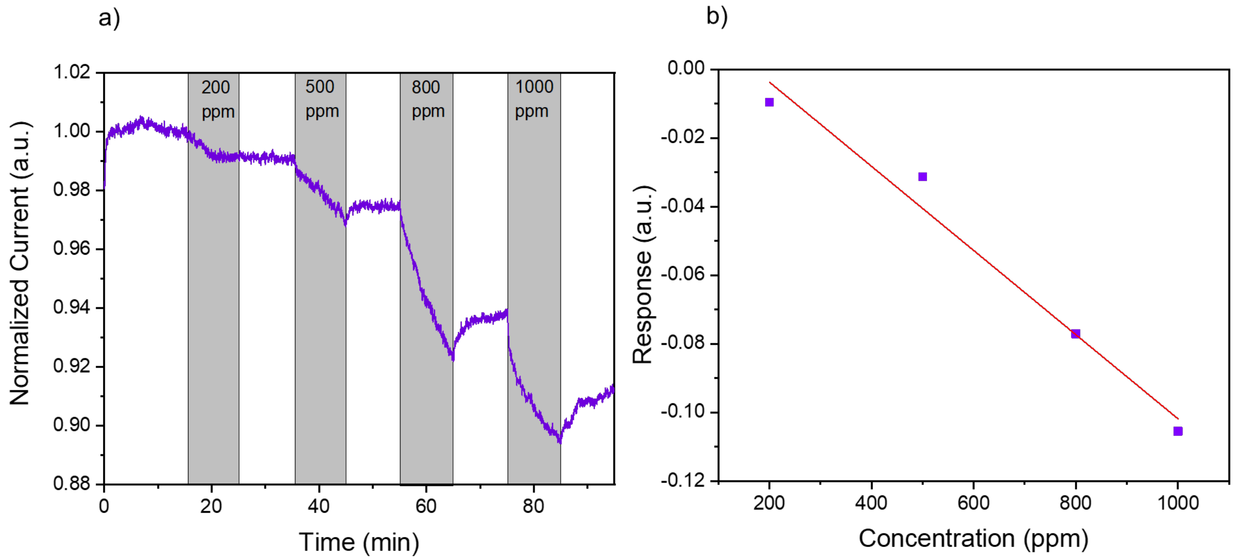

Figure S16. Chemiresistive sensing of acetone with layer-by-layer synthesized MIL-53 (Al)-NH_2 MOF on silicon chip with ox-SWCNTs. a) Normalized device current versus time during exposures to different concentrations of acetone vapors (200, 500, 800 and 1000 ppm). b) Linear calibration plot for chemiresistive sensing of acetone with the device.

Table S2. Comparison table for acetone sensors.

Material	Testing concentration range	Temperature ºC	Ref.
VNU-15 MOF	1-10 ppm	50	S1
MOF derived carbon NPs decorated α-Fe_2O_3	500 ppb-2.5 ppm	225	S2
CNTs@α-Fe_2O_3	500 ppb-100 ppm	225	S3
VO_2@ZnO hetero nanostructures	10-200 ppm	Room Temperature	S4
ZIF-8/MWCNTs resistive sensor	0.62-40 ppm	Room Temperature	S5
ZnO-BTC MOF	0.1-20 ppm	300	S6
Pd@ZIF-67 derived PdO@Co_3O_4	1-5 ppm	350	S7
Au@Co_3O_4 core-shell nanoparticles	2-10 ppm	250	S8
TiO_2@SWCNT with dehumidifier	4.8-100 ppm	Room Temperature	S9
MIL-53 (Al)-NH_2/ox-SWCNT	200-1000 ppm	Room Temperature	This work

References.

S1. Nguyen, L. H. T.; Navale, S. T.; Yang, D. H.; Nguyen, H. T. T.; Phan, T. B.; Kim, J.-Y.; Mirzaei, A.; Doan, T. L. H.; Kim, S. S.; Kim, H. W., Fe-based metal-organic framework as a chemiresistive sensor for low-temperature monitoring of acetone gas. *Sens. Actuators B: Chem.* **2023,** *388*, 133799.

S2. Zhu, L.-Y.; Yuan, K.; Li, Z.-C.; Miao, X.-Y.; Wang, J.-C.; Sun, S.; Devi, A.; Lu, H.-L., Highly sensitive and stable MEMS acetone sensors based on well-designed α-Fe2O3/C mesoporous nanorods. *J. Colloid Interface Sci.* **2022,** *622*, 156-168.

S3. Dai, M.; Zhao, L.; Gao, H.; Sun, P.; Liu, F.; Zhang, S.; Shimanoe, K.; Yamazoe, N.; Lu, G., Hierarchical assembly of α-Fe_2O_3 nanorods on multiwall carbon nanotubes as a high-performance sensing material for gas sensors. *ACS Appl. Mater. Interfaces* **2017,** *9* (10), 8919-8928.

S4. Liang, J.; Yang, R.; Zhu, K.; Hu, M., Room temperature acetone-sensing properties of branch-like VO_2 (B)@ZnO hierarchical hetero-nanostructures. *J. Mater. Sci.: Mater. Electron.* **2018,** *29* (5), 3780-3789.

S5. Homayoonnia, S.; Kim, S., ZIF-8/MWCNT-nanocomposite based-resistive sensor for highly selective detection of acetone in parts-per-billion: Potential noninvasive diagnosis of diabetes. *Sens. Actuators B: Chem.* **2023,** *393*, 134197.

S6. Du, B.; Yan, F.; Lin, X.; Liang, C.; Guo, X.; Tan, Y.; Zhen, H.; Zhao, C.; Shi, Y.; Kibet, E.; He, Y.; Yang, X., A bottom-up sonication-assisted synthesis of Zn-BTC MOF nanosheets and the ppb-level acetone detection of their derived ZnO nanosheets. *Sens. Actuators B: Chem.* **2023,** *375*, 132854.

S7. Koo, W.-T.; Yu, S.; Choi, S.-J.; Jang, J.-S.; Cheong, J. Y.; Kim, I.-D., Nanoscale PdO Catalyst Functionalized Co_3O_4 Hollow Nanocages Using MOF Templates for Selective Detection of Acetone Molecules in Exhaled Breath. *ACS Appl. Mater. Interfaces* **2017,** *9* (9), 8201-8210.

S8. Lee, H. Y.; Bang, J. H.; Majhi, S. M.; Mirzaei, A.; Shin, K. Y.; Yu, D. J.; Oum, W.; Kang, S.; Lee, M. L.; Kim, S. S., Conductometric ppb-level acetone gas sensor based on one-pot synthesized Au@ Co_3O_4 core-shell nanoparticles. *Sens. Actuators B: Chem.* **2022,** *359*, 131550.

S9. Hwang, S. I.; Chen, H.-Y.; Fenk, C.; Rothfuss, M. A.; Bocan, K. N.; Franconi, N. G.; Morgan, G. J.; White, D. L.; Burkert, S. C.; Ellis, J. E., Breath acetone sensing based on single-walled carbon nanotube–titanium dioxide hybrids enabled by a custom-built dehumidifier. *ACS Sens.* **2021,** *6* (3), 871-880.
